# SARS Coronavirus Papain-Like Protease Inhibits the TLR7 Signaling Pathway through Removing Lys63-Linked Polyubiquitination of TRAF3 and TRAF6

**DOI:** 10.3390/ijms17050678

**Published:** 2016-05-05

**Authors:** Shih-Wen Li, Ching-Ying Wang, Yu-Jen Jou, Su-Hua Huang, Li-Hsin Hsiao, Lei Wan, Ying-Ju Lin, Szu-Hao Kung, Cheng-Wen Lin

**Affiliations:** 1 Department of Medical Laboratory Science and Biotechnology, China Medical University, Taichung 404, Taiwan; violet7053@gmail.com (S.-W.L.); spirit1126@hotmail.com (C.-Y.W.); alvajou@gmail.com (Y.-J.J.); bonny6789@gmail.com (L.-H.H.); 2 Department of Biotechnology, College of Health Science, Asia University, Wufeng, Taichung 413, Taiwan; shhuang@asia.edu.tw; 3 Department of Medical Genetics and Medical Research, China Medical University Hospital, Taichung 404, Taiwan; lei.joseph@gmail.com (L.W.); yjlin.kath@gmail.com (Y.-J.L.); 4 Department of Biotechnology and Laboratory Science in Medicine, National Yang Ming University, Taipei 112, Taiwan; szkung@ym.edu.tw

**Keywords:** SARS-CoV, Toll-like receptor 7, imiquimod, TRAF3, TRAF6, Lys63-linked polyubiquitin chains

## Abstract

Severe acute respiratory syndrome coronavirus (SARS-CoV) papain-like protease (PLPro) reportedly inhibits the production of type I interferons (IFNs) and pro-inflammatory cytokines in Toll-like receptor 3 (TLR3) and retinoic acid-inducible gene 1 (RIG-I) pathways. The study investigated the inhibitory effect and its antagonistic mechanism of SARS-CoV PLPro on TLR7-mediated cytokine production. TLR7 agonist (imiquimod (IMQ)) concentration-dependently induced activation of ISRE-, NF-κB- and AP-1-luciferase reporters, as well as the production of IFN-α, IFN-β, TNF-α, IL-6 and IL-8 in human promonocyte cells. However, SARS-CoV PLPro significantly inhibited IMQ-induced cytokine production through suppressing the activation of transcription factors IRF-3, NF-κB and AP-1. Western blot analysis with anti-Lys48 and anti-Lys63 ubiquitin antibodies indicated the SARS-CoV PLPro removed Lys63-linked ubiquitin chains of TRAF3 and TRAF6, but not Lys48-linked ubiquitin chains in un-treated and treated cells. The decrease in the activated state of TRAF3 and TRAF6 correlated with the inactivation of TBK1 in response to IMQ by PLPro. The results revealed that the antagonism of SARS-CoV PLPro on TLR7-mediated innate immunity was associated with the negative regulation of TRAF3/6-TBK1-IRF3/NF-κB/AP1 signals.

## 1. Introduction

Toll-like receptor 7 (TLR7) is one of the well-known pattern recognition receptors [1], sensing single-stranded RNA viruses via the recognition of the viral RNA genome [2]. Binding of the ligand to the TLR extracellular domain causes the homodimerization of TLRs via further interaction of the intracellular Toll/interleukin-1 receptor (TIR) domain, then activates myeloid differentiation factor 88 (MyD88)-dependent and/or TIR domain-containing adaptor-inducing IFN-β (TRIF)-dependent pathways [1,3]. In the MyD88-dependent pathway, the complex of MyD88 with activated TLR dimers, except for TLR3, recruits IL-1 receptor-associated kinase (IRAK)-4, then leads IRAK4 to activate other members of the IRAK, like IRAK1 and IRAK2. The activated IRAKs directly interact with TNF receptor-associated factor (TRAF) 6 (E3 ubiquitin ligase) plus E2 ubiquitin-conjugating enzymes Ubc13 and Uev1A, resulting in Lys63-linked ubiquitination of TRAF6, IRAKs and TGF-β-activated kinase 1 (TAK1) [4]. In the TRIF-dependent pathway, activated TLR3 directly binds TRIF, resulting in the activation of TRIF connecting with TRAF3 to turn on TANK-binding kinase 1 (TBK1) and IKKs for IRF3/7 phosphorylation [4,5]. Alternatively, TRIF associates with RIP1 and then forms the complex along with TRAF6 for activation of TAK1. Ubiquitin-activated TAK1 phosphorylates mitogen-activated protein kinases (MAPKs) and IκB-kinases (IKKs), initiating AP-1, NF-κB and IRF3/7 signaling on the production of cytokines, chemokines and type I interferons (IFNs).

Severe acute respiratory syndrome (SARS)-associated coronavirus (SARS-CoV) causes the pro-inflammatory cytokine storms, recruitment of immune responder cells into the lungs, acute respiratory distress syndrome (ARDS) and even lung fibrosis in the late phase [6,7]. Among 14 ORFs encoded by SARS-CoV [8,9], ORF1a/ORF1ab is the biggest one encoding polyprotein replicases 1a and 1ab, primarily involved in SARS-CoV replication. Specifically, ORF1a-encoded papain-like protease (PLPro) and 3C-like protease (3CLpro) process *cis*- and *trans*-cleavage activities on replicases 1a and 1ab for creating 16 nonstructural (NS) proteins (termed NS 1 through NS16). PLPro, recognizing a consensus motif LXGG as a de-ubiquitinating/de-ISGylating enzyme [10,11,12,13], exhibits the antagonistic activities of type I interferon (IFN). PLPro directly interacts with IFN regulatory factor 3 (IRF-3) to block IRF-3 phosphorylation and nuclear translocation [14,15]. Conversely, another study indicated that type I IFN antagonism of PLPro is not correlated to a direct interaction of PLPro with IRF-3 or affecting the phosphorylation of IRF3, but PLPro suppresses the NF-κB signaling pathway by preventing IκBα degradation [15]. Recent studies demonstrate that PLPro disrupts the stimulator of interferon gene (STING)-mediated signaling and then negatively regulates type I IFN induction [16,17]. PLPro physically interacts with the STING-TRAF3-TBK1 complex, reducing the ubiquitinated forms of STING, RIG-I, TRAF3, TBK1 and IRF-3. Similarly, de-ubiquitinating enzyme cylindromatosis (CYLD) inhibits NF-κB signaling via de-ubiquitination and inactivation of TNFR-associated factor 2 (TRAF2) and TRAF6 [18,19]; de-ubiquitinating protease A20 inhibits NF-κB activation induced by Toll-like receptor 4 (TLR4) via removing K63-linked polyubiquitin chains of TRAF6 [20]. PLPro interacts with above key regulators of TLR signal pathways; thus, characterizing the antagonistic mechanisms of TLR signal pathways by SARS-CoV PLPro could provide valuable insights into SARS pathogenesis.

SARS-CoV-specific GU-rich ssRNA has been demonstrated to be recognized by TLR7, establishing a link with the induction of pro-inflammatory cytokines TNF-α, IL-6 and IL-12 [21]. SARS-CoV infection rapidly activates TLR7 signaling in plasmacytoid dendritic cells; however, some SARS-CoV proteins subsequently inhibit and/or modulate type I IFN responses in plasmacytoid dendritic cells [22]. Therefore, the effect of the unique SARS-CoV proteins on the activation of the TLR7 signaling pathway is noteworthy to elucidate.

This study assesses possible effects of PLPro on TLR7 signaling pathways and the production of type I IFNs and pro-inflammatory cytokines. In this study, PLPro-expressing and vector control cells were treated with TLR7 agonist (imiquimod (IMQ)) and then examined on IRF3, STAT1, NF-κB, p38 MAPK and c-Jun regulation using dual luciferase reporters, Western blotting and real-time PCR assays. Analysis of ubiquitin-modified proteins reveals that the change in ubiquitination of TRAF3 and TRFA6 played the crucial role in the antagonistic mechanisms of TLR7-mediated type I IFN induction and NF-κB activation by PLPro.

## 2. Results

### 2.1. PLPro Suppressed TLR7 Agonist-Induced Production of Type I IFNs in Human Promonocytes

**Figure 1:**
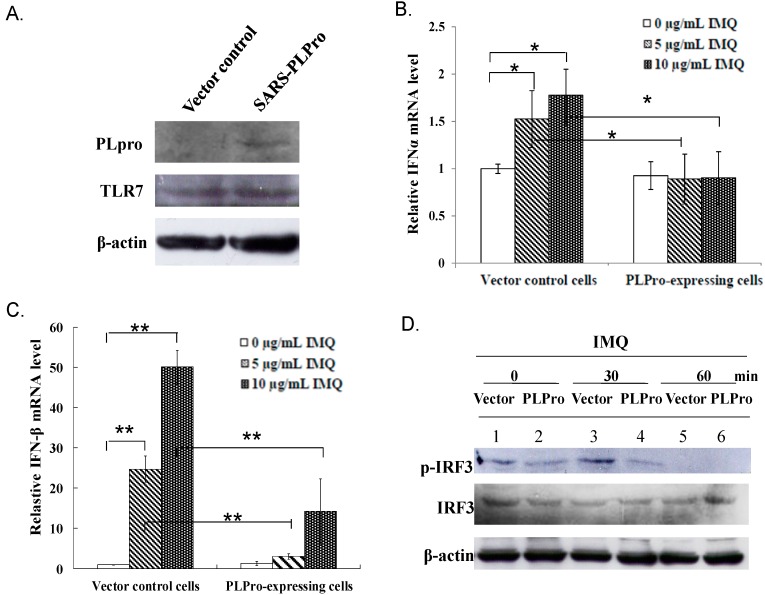
Effect of SARS-CoV PLPro on TLR7 agonist-induced production of type I IFNs via IRF3 signaling. The expression level of PLPro and TLR7 in the vector control and PLPro-expressing cells was analyzed using Western blot assay (**A**). Both types of transfected cells were treated with or without imiquimod (IMQ) for 4 h, and then, their mRNA levels of IFN-α and IFN-β were measured by quantitative PCR. Relative mRNA levels of IFN-α (**B**) and IFN-β (**C**) were normalized by GAPDH mRNA, presented as a relative ratio. To determine IRF3 activation, the lysates were also analyzed using Western blot with anti-phospho-IRF3 antibodies (**D**). * *p*-Value < 0.05; ** *p*-value < 0.01 by Student’s *t*-test.

**Figure 2:**
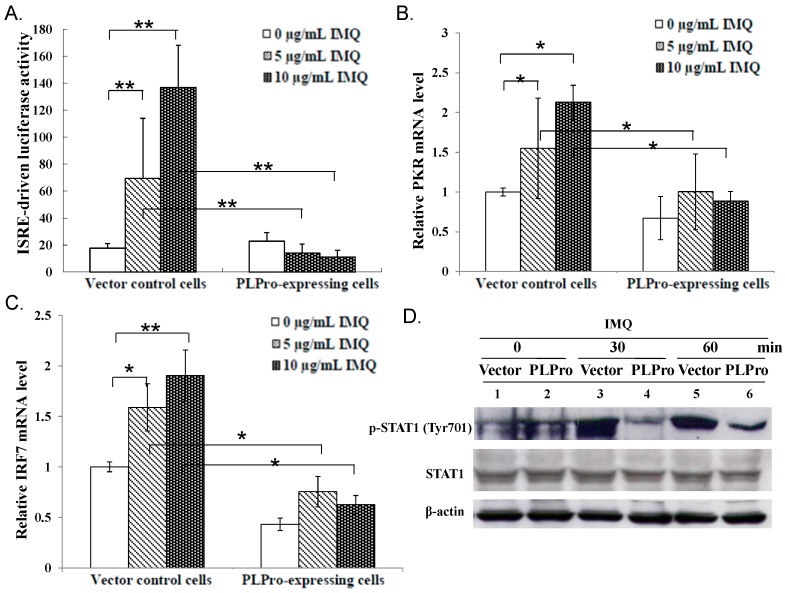
Inhibitory effect of SARS-CoV PLPro on TLR7 agonist-induced activation of type I IFN signaling. ISRE-driven luciferase reporter activity and the mRNA levels of PKR and IRF7 were determined 4 h post-IMQ treatment. ISRE-driven firefly luciferase activity was normalized by Renilla luciferase activity (**A**). Relative mRNA levels of PKR (**B**) and IRF7 (**C**) were normalized by GAPDH mRNA, presented as a relative ratio. In addition, the activated status of STAT1 was examined using Western blot with anti-phospho-STAT1 (Tyr701) antibodies (**D**). * *p*-Value < 0.05; ** *p*-value < 0.01 by Student’s *t*-test.

To examine whether SARS-CoV PLPro modulates the TLR7 signaling pathway, stable transfected promonocyte cells expressing PLPro and vector control cells were established, treated with TLR7 agonist (imiquimod (IMQ)), then further analyzed for activation of type I IFN production (Figure 1). Western blot analysis showed a 60-kDa band recombinant PLPro protein in transfected cells, but not controls, as well as the similar expression level of TLR7 in both types of cells (Figure 1A), demonstrating that SARS-CoV PLPro was stably expressed in human promonocyte cells that did not alter the TLR7 expression. Quantitative real-time PCR signified TLR7 agonist treatment stimulating higher transcriptional levels of IFN-α and IFN-β in vector controls than in PLPro-expressing cells (Figure 1B,C). Western blotting analysis of both of these cells indicated that IRF3 phosphorylation in vector control cells reached a maximum level at 30 min, but disappeared at 60 min after IMQ treatment. Importantly, PLPro inhibited IMQ-induced activation of IRF3 phosphorylation since no significant change in IRF3 phosphorylation was detectable at 30 min after IMQ treatment compared to non-treatment (Figure 1D). To assess the functional activity of IFN-α and IFN-β induced by TLR7 agonist, the interferon-stimulated response element (ISRE) reporter of the dual-luciferase assay was further performed (Figure 2A). The TLR7 agonist dose-dependently triggered the ISRE promoter activity in vector control cells, but not in PLPro-expressing cells (Figure 2A). Quantitative real-time PCR signified that the TLR7 agonist dose-dependently upregulated the IFN-stimulated genes (ISGs), including PKR and IRF7, in vector controls, but not PLPro-expressing cells (Figure 2B,C). In addition, TLR7 agonist-induced activation of STAT1 was delayed and suppressed by SARS-CoV PLPro (Figure 2D). Results revealed that PLPro suppresses TLR7 agonist-induced production of type I IFNs through inhibiting the IRF-3 activation in the TLR7 signaling pathway.

### 2.2. Inhibition of TLR7 Agonist-Induced Pro-Inflammatory Cytokines by SARS-CoV PLPro

**Figure 3:**
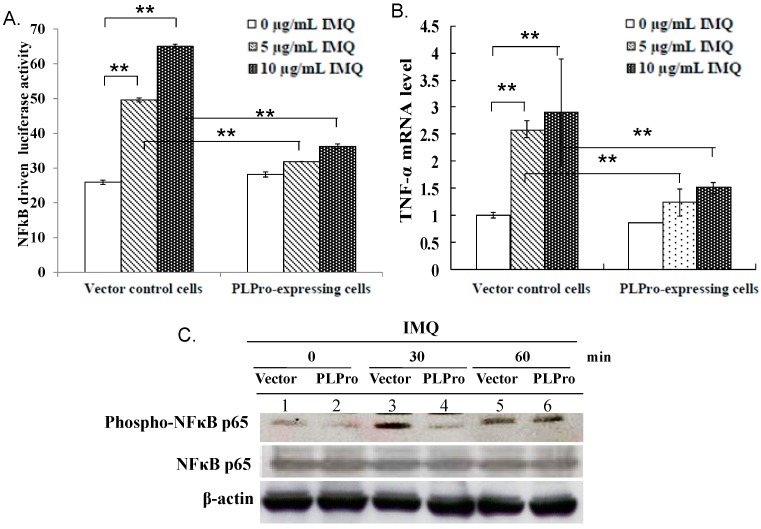
Inhibition of IMQ-induced TNF-α production via NF-κB signaling by SARS-CoV PLPro. Both types of cells were treated with(out) IMQ for 4 h, and then, NF-κB-driven luciferase reporter activity and the TNF-α mRNA level were determined using the dual luciferase reporter assay (**A**) and quantitative PCR (**B**), respectively. For determining NF-κB activation, the lysates were also analyzed using Western blot with anti-phospho-NF-κB p65 antibodies (**C**). ** *p*-Value < 0.01 by Student’s *t*-test.

**Figure 4:**
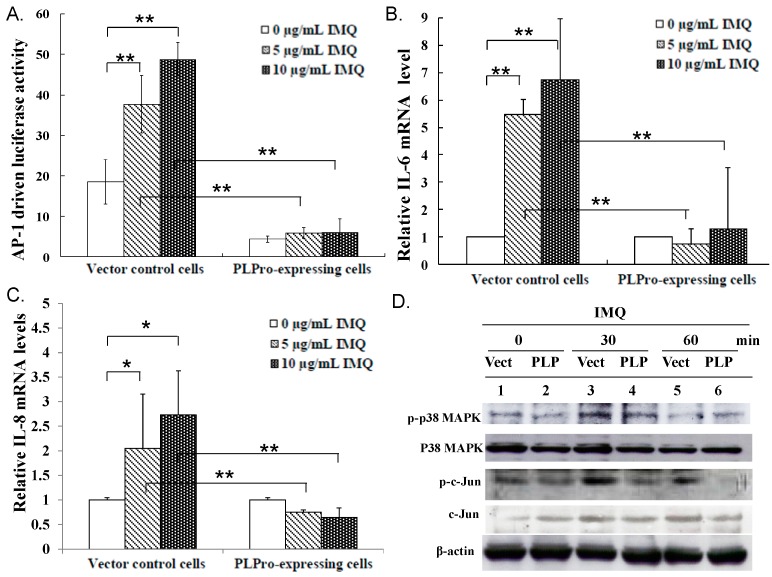
Detection of IMQ-induced AP-1-mediated production of IL-6 and IL-8 in the vector control and PLPro-expressing cells. AP-1-driven firefly luciferase activity was normalized by Renilla luciferase activity (**A**). Relative mRNA levels of IL-6 (**B**) and IL-8 (**C**) were normalized by GAPDH mRNA, presented as a relative ratio. In addition, the activated status of p38 MAPK and AP-1 was examined using Western blot with anti-phospho-p38 MAPK and anti-phospho-c-Jun antibodies (**D**). * *p*-Value < 0.05; ** *p*-value < 0.01 by Student’s *t*-test.

Besides type I IFN production, we checked whether the TLR7 agonist upregulates pro-inflammatory cytokines. The effect of SARS-CoV PLPro on the activation of NF-κB and AP1 promoters, as well as the production of pro-inflammatory cytokines (IL-6, IL-8 and TNF-α) was further characterized via dual-luciferase reporter assay and SYBR green real-time PCR (Figure 3 and Figure 4). Results indicated that SARS-CoV PLPro inhibits TLR7 agonist-induced activation of NF-κB and AP1 promoters, which was associated with reducing the stimulated upregulation of IL-6, IL-8 and TNF-α mRNA (Figure 3A,B and Figure 4A–C). To explore the inhibitory effect of PLPro on NF-κB and AP1 signals, the levels of NF-κB p65, p38 MAPK and c-Jun phosphorylation were subsequently detected in responses to TLR7 agonist using Western blotting (Figure 3C and Figure 4D, respectively). Western blotting demonstrated that PLPro suppressed TLR7 agonist-induced phosphorylation of NF-κB, p38 MAPK and c-Jun compared to those in the vector control at 30 min post-treatment. The results demonstrated that SARS-CoV PLPro suppressed TLR7-induced cytokines through decreasing the phospho-NF-κB p65, p38 MAPK and c-Jun, resulting in suppressing of the NF-κB and AP-1 signaling pathway.

### 2.3. Ubiquitin Removal of TRAF3 and TRAF6 Responsible for the TLR7 Antagonism of SARS-CoV PLPro

**Figure 5:**
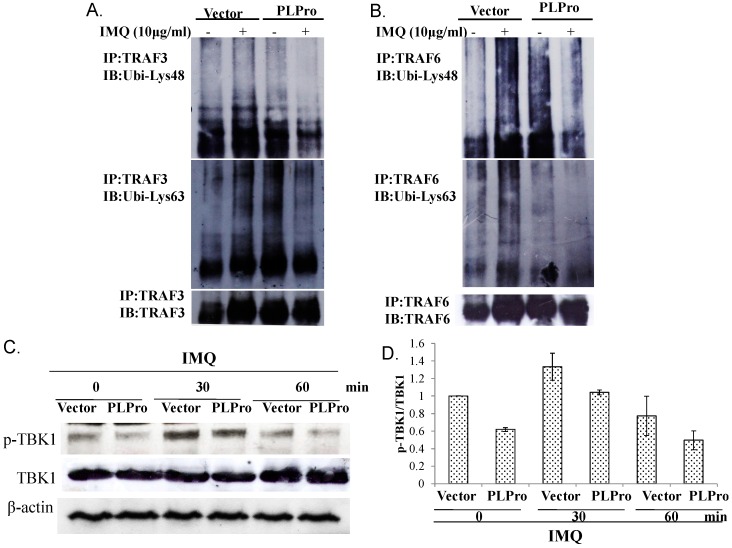
Detecting the Lys48- and Lys63-linked ubiquitination of TRAF3 and TRAF6 measured by the immunoprecipitation assay. Vector control and PLPro-expressing cells were treated with(out) IMQ for 1 day; cell lysates were immunoprecipitated with anti-TRAF3 (**A**) or anti-TRAF6 (**B**) followed by Western blotting probed with either anti-Lys48 ubiquitin or anti-Lys63 ubiquitin antibodies. Phospho-TBK1 levels were detected by Western blot (**C**). The relative band intensity of phospho-TBK1 was normalized by TBK1, compared to the mock vector control cells, and quantified using ImageJ based on triplicate replicates of each experiment (**D**).

To examine the inhibitory mechanism of TLR7 antagonism by SARS-CoV PLPro, differential profiles of ubiquitin-conjugated proteins in the vector control and PLPro-expressing cells in the absence or presence of IMQ were analyzed using immune-precipitation (Figure 5A,B). Because Lys63-linked auto-ubiquitination of TRAF is required for its E3 ubiquitin ligase activity and SARS-CoV PLPro proceeds the de-ubiquitination enzymatic activity, the ubiquitination status of TRAF3 and TRAF6 was subsequently analyzed for whether they were involved in the TLR7 signaling pathway induced by IMQ (Figure 5). The immunoprecipitation assay indicated that PLPro reduced Lys48- and Lys63-linked poly-ubiquitination of TRAF3 and TRAF6 induced by IMQ (Figure 5A,B, Lane 2 *vs.* 4). Subsequently, Western blotting analysis indicated that IMQ-induced activation of TBK1 was suppressed in PLPro-expressing cells (Figure 5C, Lane 3 *vs.* Lane 4; Figure 5D). Therefore, the results indicated that PLPro reduced Lys63-linked poly-ubiquitination of TRAF3 and TRAF6, which correlated with the activation of TBK1, p38 MAPK, NF-κB and IRF3 in the TLR7 signaling pathway.

## 3. Discussion

This study was the first report in which SARS-CoV PLPro showed antagonistic activity against TLR7 agonist-induced production of IFN-α, IFN-β, TNF-α, IL-6 and IL-8 via reducing TLR7 agonist-induced phosphorylation of transcription factors IRF3, NF-κB and c-Jun (Figure 1, Figure 3 and Figure 4). TLR7 agonist-induced IFN-β stimulated the transcriptional activity of STAT1 to upregulate the expression of ISGs, such as PKR and IRF7, in vector control cells (Figure 2). However, there was no significant response of STAT1-mediated signaling due to the inhibitory effect of PLPro on the production of TLR7 agonist-induced IFN-β (Figure 2). The results were constant with previous reports in that SARS-CoV PLPro inhibited TLR3- and RIG-I-mediated production of type I interferons and proinflammatory cytokines via inactivating the transcription factors IRF3 and NF-κB [15,23].

This study proposed TRAF3 and TRAF6 as the crucial regulators of PLPro antagonism via modification of the profiles of ubiquitin-conjugate proteins in the vector control and PLPro-expressing cells; thus, the ubiquitin linkage status of TRAF3 and TRAF6 was analyzed post-treatment with(out) IMQ using immunoprecipitation (Figure 5). Immunoprecipitation assays indicated that PLPro decreased the Lys63-linked polyubiquitin chains of TRAF3 and TRAF6, causing the decrease in the E3 ubiquitin ligase activity of TRAF3 and TRAF6, which correlated with lower levels of TBK-1 phosphorylation in PLPro-expressing cells than vector control cells in response to IMQ. PLPro has been reported to upregulate the ubiquitin-conjugating enzyme E2-25k and proteasome subunit alpha type 5, which resulted in the increase of ERK1 poly-ubiquitination in PLPro-expressing cells as the type I IFN antagonistic mechanism of SARS PLPro via the degradation of ERK1 [24,25]. The high ubiquitination level of TRAF3 and TRAF6 in PLPro-expressing cells (Figure 5A,B) might be responsible for the activation of the ubiquitin proteasome pathway, as demonstrated in our prior reports [24,25]. The finding was also similar to previous reports in that the inhibitory effect of SARS-CoV PLPro on the inactivation of the transcription factors IRF3 and NF-κB has been demonstrated to correlate with PLPro-reduced ubiquitination of RIG-I, STING TRAF3, TBK1 and IRF3 in the TLR3 and RIG-I pathways [16,17]. These previous reports proposed an antagonistic mechanism of SARS-CoV PLPro, including the inhibition of STING-MAVS-TBK1/IKKε and STING-TRAF3-TBK1 signaling in the TLR3 and RIG-I pathways. Since TBK1 has been suggested to activate protein phosphatases [26], no detectable level of IRF3 phosphorylation, as well as the decrease of NF-κB p65, p38 MAPK and c-Jun phosphorylation in the vector control and PLPro-expressing cells at 60 min post-IMQ treatment (Figure 1D and Figure 3C) could be due to a the negative feedback of the TBK1-IRF3/NF-κB pathways. Therefore, we propose that the inhibition of TRAF3/6-TBK1-IRF3/NF-κB/AP1 signals by PLPro is responsible for the antagonistic mechanism of SARS-CoV PLPro against TLR7-medaited production of type I IFNs and pro-inflammatory cytokines.

## 4. Materials and Methods

### 4.1. Cell Line and Western Blot Assay

SARS-CoV PLPro-expressing and empty vector control promonocyte cell lines were established, as described in our previous reports [24,25]. Both stably-transfected cell lines grew in RPMI-1640 medium containing 10% FBS and 800 μg/mL of Geneticin^®^ Selective Antibiotic (G418 Sulfate). The expression level of PLPro and TLR7 in both stable cell lines was analyzed using Western Blotting with anti-PLPro mouse sera and anti-TLR7 mAb. To monitor the activation of the IMQ-induced TLR7 signaling pathway, PLPro-expressing and vector control cells were treated with 10 μM IMQ (InvivoGen, San Diego, CA, USA) and harvested 0, 30 and 60 min post-treatment. Lysate from IMQ-treated cells was used for the Western blotting assay; the resulting blots were probed with specific primary antibodies against IRF3, phosphor-IRF3, STAT1, phospho-STAT1(Tyr701), NF-κB p65, phospho-NF-κB p65, p38 MAPK, phospho-p38 MAPK, c-Jun, phospho-c-Jun, TBK1, phospho-TBK1, TRAF3, TRAF6 and anti-β-actin mAb (Abcam, Cambridge, UK). Immune complexes were detected with horseradish peroxidase-conjugated secondary antibodies, followed by enhanced chemiluminescence detection (Amersham Pharmacia Biotech, Piscataway, NJ, USA).

### 4.2. Signaling Pathway Assays with the Dual Luciferase Reporter System

*Cis-*reporter plasmids pISRE-Luc, pAP-1-Luc and pNF-κB-Luc were used to examine the firefly luciferase activity driven from each *cis*-acting transcriptional element. SARS-CoV PLPro-expressing and vector control cells were transfected with the indicated *cis*-reporter plasmid, plus internal control reporter pRluc-C1 (BioSignal Packard, Montréal, QC, Canada) using GenePorter reagent. After 4 h of incubation with or without IMQ, the activity of experimental firefly luciferase and control Renilla luciferase was measured by the dual Luciferase Reporter Assay System (Promega, Madison, WI, USA) and the Clarity™ Luminescence Microplate Reader (BioTek Instruments, Bad Friedrichshall, Germany) [27].

### 4.3. Quantitative Expression Analysis of Type I IFNs and Pro-Inflammatory Cytokines Using Real-Time PCR

PLPro-expressing or empty vector cells were incubated for 4 h in the presence or absence of IMQ and harvested for isolation of total RNAs by a PureLink Micro-to-Midi Total RNA Purification System Kit (Invitrogen, Carlsbad, CA, USA), following quantification of gene expression by a two-step real-time PCR using SYBR Green I. First, cDNA was synthesized from 1 μg total RNA, using oligonucleotide d(T) primer and a SuperScript III reverse transcriptase kit (Invitrogen). Each real-time PCR reaction contained 5 μL of a cDNA mixture, 1 μL of primer pair (200 nM) and 12.5 μL Smart Quant Green Master Mix with Dutp and ROX (Protech, Vancouver, WA, USA). Primer pairs included (1) 5′-CAACCAGCGGTTGACTTTTT-3′ and 5′-ATCCAGGAAGGCAAACTGAA-3′ for PKR; (2) 5′-GTGAGGAAATACTTCCAAAGAATCAC-3′ and 5′-TCTCATGATTTCTGCTCTGACAA-3′ for IFN-α; (3) 5′-AACTGCAACCTTTCGAAGCC-3′ and 5′-TGTCGCCTACTACCTGTTGTGC-3′ for IFN-β; (4) 5′-CGCGGCACTAACGACAGGCGAG-3′ and 5′-GCTGCCGTGCCCGGAATTCCAC-3′ for IRF7; (5) 5′-CTTCTCCTTCCTGATCGTGG-3′ and 5′-GCTGGTTATCTCTCAGCTCCA-3′ for TNF-α; (6) 5′-GATGGATGCTTCCAATCTGGAT-3′ and 5′-AGTTCTCCATAGAGAACAACATA-3′ for IL-6; (7) 5′-CGATGTCAGTGCATAAAGACA-3′ and 5′-TGAATTCTCAGCCCTCTTCAAAAA-3′ for IL-8; and (8) 5′-AGCCACATCGCTCAGACAC-3′ and 5′-GCCCAATACGACCAAATCC-3′ for glyceraldehyde-3-phosphate dehydrogenase (GAPDH). Real-time PCR was performed as described in our previous reports [23,24]. The amplification and detection of specific products were conducted in an ABI Prism 7900 sequence detection system (PE Applied Biosystems, Carlsbad, CA, USA). Relative changes in the mRNA level of indicated genes were normalized relative to GAPDH mRNA.

### 4.4. Immunoprecipitation Assays

To detect ubiquitination of TRAF3 and TRAF6, lysates from PLPro-expressing or vector cells with or without IMQ were collected, incubated with specific monoclonal antibodies anti-TRAF3 or anti-TRAF6 for 4 h at 4 °C and then followed by the addition of protein A-Sepharose beads and an additional 2 h of incubation. After centrifugation, pellets were washed four times with NET buffer (150 mM NaCl, 0.1 mM EDTA, 30 mM Tris-HCl, pH 7.4); immunoprecipitated proteins were dissolved in 2× SDS-PAGE sample buffers without 2-mercaptoethanol, boiled for 10 min, separated by SDS-PAGE and then transferred to nitrocellulose. Resulting blots were blocked with 5% skim milk, reacted with specific monoclonal antibodies anti-TRAF3, anti-TRAF6, anti-Lys48- or anti-Lys63-linked ubiquitin and then followed by enhanced chemiluminescence detection.

### 4.5. Statistical Analysis

Data were from three independent experiments; error bars represent the standard error of the mean. Chi-square and Student’s *t*-test were used to analyze all data, with statistical significance between types of cells noted at *p* < 0.05.

## 5. Conclusions

In summary, SARS-CoV PLPro inhibits TLR7-mediated signaling, leading to reducing of the cytokine production during antiviral responses. PLPro diminishes Lys63-linked ubiquitination of TRAF3 and TRAF6 and then inactivates their downstream molecules, such as kinases (TBK1 and p38 MAPK) and transcription factors (IRF3, NF-κB and AP-1). The results let us conclude that SARS-CoV PLPro negatively regulates the TRAF3/6-TBK1-IRF3/NF-κB/AP1 signals in TLR7-mediated antiviral and inflammatory responses.
